# Testing anxiety in undergraduate medical students and its correlation with different learning approaches

**DOI:** 10.1371/journal.pone.0210130

**Published:** 2019-03-13

**Authors:** Christine Cipra, Brigitte Müller-Hilke

**Affiliations:** Institute of Immunology, University Medical Center Rostock, Rostock, Germany; Stockholms Universitet, SWEDEN

## Abstract

**Objectives:**

Undergraduate medical students experience a considerable amount of stress and anxiety due to frequent exams. The goal of the present study was to examine the development of exam related anxiety and to test for a correlation between anxiety and learning approaches.

**Methods:**

A whole class of 212 medical students was invited to participate in the study. During their first term, trait anxiety and learning approaches were assessed by use of the state-trait-anxiety inventory (STAI-T) and the approaches-and-study-skills-inventory-for-students (ASSIST), respectively. Acute state anxiety was assessed twice in the course of the second term. To that extent, the STAI-S in combination with measuring salivary cortisol were employed immediately before two oral anatomy exams.

**Results:**

Our most important results were that a surface learning approach correlated significantly with anxiety as a trait and that students with a predominantly strategic approach to learning were the least anxious yet academically most successful.

**Conclusion:**

As surface learners are at risk of being academically less successful and because anxiety is a prerequisite for burn-out, we suggest that medical faculties place particular emphasis on conveying strategies for both, coping with stress and successful learning.

## Introduction

Education in medical school is demanding and aims at graduating knowledgeable, skillful and mentally healthy physicians that will tend to their patients’ needs with empathy and professionalism. However, studies show that licensed medical doctors experience high levels of stress related anxiety and burn-out that not only endanger professionalism but also correlate with medical incidents [1, 2]. At close inspection, students in the early phase of medical school already reveal a decline in mental health and mental health will remain poor throughout their training [3, 4]. Reasons for distress are manifold and include adjustment to the medical school environment and exposure to death and human suffering, but also imbalances between efforts invested and rewards received combined with anxiety resulting from numerous exams and academic challenges [5, 6]. While for some, stress may be a motivator for academic performance, it may arouse feelings of fear, incompetence or even anger in others [4]. We therefore grew interested in a connectedness between the individual learning approaches that will result in academic performance on the one hand and medical students’ anxiety on the other.

According to Spielberger, anxiety differentiates into a permanent personality trait and an acute state as experienced immediately before an exam [7]. Both have been reported to result from non-academic–e.g. gender—as well as academic–e.g. school grade—risk factors [8]. Moreover, anxiety was shown to increase during medical school education [9] and to be fueled by a high degree of perfectionism among medical students [10]. As success in a demanding academic environment and the distress resulting from fear of failure are by necessity linked to learning, we were intrigued by the students’ learning approaches. A surface learning approach applies to passive learners who rely on rote learning and see learning as coping with tasks so they can pass assessment. By contrast, students with a deep approach to learning are intrinsically interested and enjoy learning, they will seek to understand meaning and have a genuine curiosity in the subject. Yet other students may use both deep and surface approaches, balancing the time at hand, the efforts considered adequate to invest and the goals defined. This latter approach is referred to as strategic [11, 12]. And even though there may be cultural preferences to the various learning approaches [13], Western medical students pursuing deep or strategic approaches were shown to achieve highest academic success whereas the surface approach correlates with poorer outcomes [14]. We thus set out to investigate whether individual learning approaches could be linked to anxiety. To that end, we addressed first year medical students and assessed in an exploratory study their individual learning approaches, their levels of anxiety as a permanent personality trait as well as acute anxiety related to exams. In parallel we monitored their academic achievements and asked if exam related anxiety increased throughout the term or directly correlated with the learning approach.

## Methods

### Participant recruitment

This exploratory study included students in their first undergraduate year at the Medical School of Rostock. During a compulsory course in the first term, the whole class of 212 students was informed about the study and invited to participate. Participation was voluntary and written informed consent was collected before the study began. Written informed consent included monitoring of academic achievements and assessment of school leaving grades. As an incentive to participate, the students were promised to obtain their personal results at the end of data collection. The study was approved by the Ethics Committee of the University Medical Center Rostock (ref. A 2016–0186).

### Study design

As acute anxiety evoked by oral exams was shown to be worse than anxiety evoked by written ones [6], we concentrated on the former and decided on the second term of the first preclinical year for the present study. In this term, the students need to pass four oral anatomy exams at intervals of three weeks, each. Failing one exam implicates a retest in the following week and consequently leaves only two weeks to prepare for the next exam. In order to assess acute anxiety, we chose the first and the third anatomy exams as these are considered equally demanding by the students. The first exam covers the upper limb and torso, while the third requires knowledge of the lower limbs and pelvic.

The study design is summarized in Table 1 and includes 5 time points (T0 –T4). T0 took place during the very first term and served to assess the students’ school leaving grades as well as trait anxiety and learning approaches via validated questionnaires. T1 and T3 served to assess acute exam-realted anxiety and took place 15 minutes before the first and the third oral anatomy exam, respectively. At these time points, the students completed a questionnaire evaluating acute anxiety and provided a salivary sample to measure cortisol. T2 and T4 served to assess cortisol baselines and took place 24 hours later than T1 and T3, respectively.

**Table 1:** Study design.

First Term	Second Term			
T0	T1	T2	T3	T4
	15 min before	1 day after	15 min before	1 day after
	oral exam	oral exam	oral exam	oral exam
ASSIST^1^				
STAI-T^2^	STAI-S^3^		STAI-S^3^	
	salivary cortisol	salivary cortisol*	salivary cortisol	salivary cortisol**

T1 –T4 during second term

*taken at exact time of day as T1 salivary cortisol

**taken at exact time of day as T3 salivary cortisol

^1^Approaches and Study Skills Inventory for Students

^2^State Trait Anxiety Inventory-Trait

^3^State Trait Anxiety Inventory-State

### Data collection tools

The State Trait Anxiety Inventory-Trait (STAI-T) and–State (STAI-S) were used in their German versions to assess anxiety as a trait and as an acute state immediately before an exam, respectively [15, 7]. The STAI is considered the gold standard for measuring anxiety and stress [16]. Both, STAI-T and STAI-S consist of 20 items with four points Likert scales, each. Scores thus range between 20, indicating a low level of anxiety and 80, indicating a high level. We decided to utilize the STAI for two reasons: first, the time for completion varies from three to six minutes and is therefore short enough to be completed before an exam and second, anxiety as a trait and as an acute state can directly be compared.

Salivary cortisol was collected into a Sarstedt-Salivette (Sarstedt, Nümbrecht, Germany) before performing an electro-chemiluminiscence immunoassay (ELICA) on an Elecsys Cortisol II (Roche Diagnostics, Basel, Switzerland).

Learning approaches were assessed trough the ASSIST questionnaire in its German version [17]. The ASSIST consists of 52 items, which can be answered with five points Likert scales. For evaluation, 13 categories are formed and summarized into the 3 main approaches deep, strategic and surface.

### Data analyses

Parametric and non-parametric methods were used to analyze data sets following Gaussian and non-Gaussian distribution, respectively. Fisher’s exact tests were performed to compare the percentages of women among the study group and the rest of the cohort. Friedman tests were performed for monitoring the levels of self-reported anxiety at T0, T1 and T3. Salivary cortisol levels were compared between T1 and T2 and T3 and T4, respectively performing Wilcoxon matched-pairs signed-rank tests. Students t-tests and Mann-Whitney U-tests were performed for the gender specific analyses. Spearman rank correlation analyses were used to determine correlation coefficients between STAI and Learning Approaches, STAI and cortisol or cortisol and learning approaches, respectively. Comparisons between learning approaches were performed via Kruskal-Wallis tests and ANOVA, respectively. P values < 0.05 were considered statistically significant. All statistical tests were carried out using IBM SPSS Statistics 22.

## Results

### Study cohort

Out of a total of 212 medical students enrolled in this term, 138 (65%) students volunteered to participate in the present study. However, only 98 students (46%) completed all anxiety and cortisol measurements and were included in subsequent analyses. The mean age of this study cohort was 20.75 years and was thus comparable to the rest of the class. Of note, the percentage of women in this study cohort was 74.5% (73/98), compared to 64.6% (25/98) in the rest of the class (p = 0.0197), indicating statistically significant differences. Of the 98 study participants, 84 had also completed the ASSIST-questionnaire (for the complete data set see S1 Table).

### Exam related anxiety was comparable throughout the term

**Fig 1:**
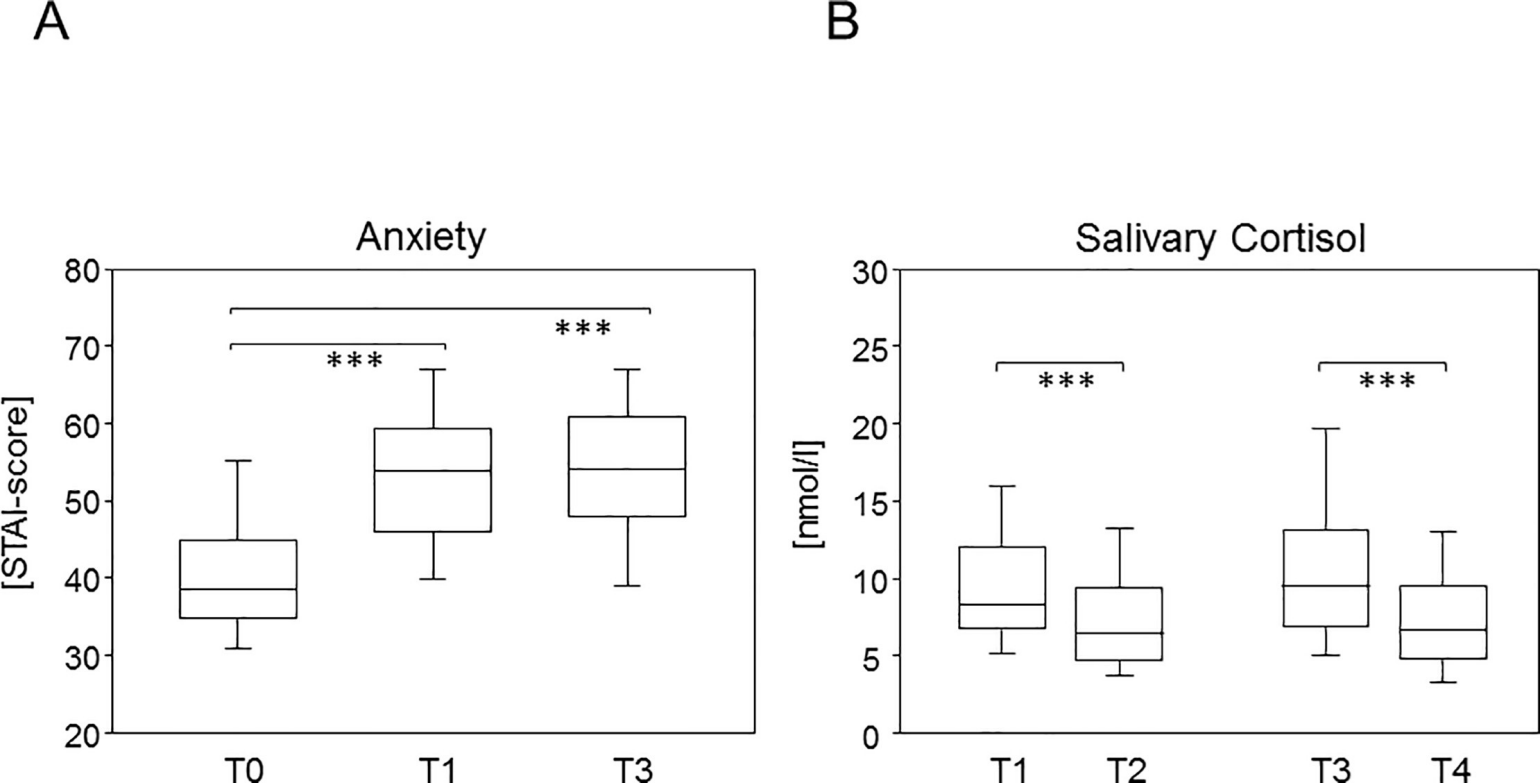
Self-reported anxiety was comparable throughout the term and was confirmed by a rise in the sympathetic stress parameter cortisol. (A) Boxplots compare the scores for trait anxiety (T0) and acute exam related anxiety at T1 and T3. Asterisks denote significant increases in anxiety before the exams. The significance levels resulting from Friedman Tests were *** for p-values < 0.0001. (B) Boxplots show salivary cortisol concentrations immediately before oral examinations (T1 and T3) and 24 hours later for determination of baseline levels (T2 and T4). Asterisks denote significant differences resulting from Wilcoxon matched-pairs signed-rank tests. *** indicate p-values < 0.0001.

Self-reported anxiety scores were lowest at the beginning of the first term (T0) when exams still lay ahead. The median STAI score at T0 was 38.5 (Fig 1A). However, anxiety significantly increased to a median score of 53.9 immediately before the first oral anatomy exam at T1. Of note, the median STAI score at T3 was 54, indicating no further increase between the first and the third anatomy exam. Self-reported anxiety was confirmed by a rise in the sympathetic stress parameter cortisol. Fig 1B shows median salivary cortisol concentrations of 8.37 and 9.53 nmol/l immediately before the oral exams at T1 and T3, respectively. Due to the circadian rhythm of cortisol levels, reference measures (T2 and T4) were taken at the exact time of day as T1 and T3 and resulted in medians of 6.46 and 6.69 nmol/l for T2 and T4, respectively [18]. Wilcoxon matched-pairs signed-ranks tests showed significant 1.2 and 1.4-fold drops of salivary cortisol one day after the oral exams. And even though this drop was slightly larger for the oral exam at T3, the difference between both oral exams was insignificant. We therefore did not observe an increase in exam related anxiety over the course of the term, neither self-reported nor assessed as a sympathetic stress parameter.

### Female students showed higher exam related anxiety

We also observed no gender-specific difference between the levels of trait anxiety (STAI-T) at the beginning of term one. However, there was a significantly higher self-reported anxiety (STAI-S) immediately before oral anatomy exams in female compared to male students (at T1: M = 54.7, SD = 10.0 (Females) and M = 48.9, SD = 9.2 (Males) conditions, t (96) = 2.57 p = 0.012; at T3: M = 55.5, SD = 10.4 (Females) and M = 49.9, SD = 8.8 (Males) conditions, t (96) = 2.43 p = 0.017). And while Mann-Whitney tests did neither reveal gender specific differences for salivary cortisol at baseline time points (T2: Mdn (Females) = 6.42 nmol/l; Mdn (Males) = 6.49 nmol/l; U = 874.5, p = 0.7599 T4: Mdn (Females) = 6.46 nmol/l; Mdn (Males) = 7.86 nmol/l; U = 717.5, p = 0.113), nor immediately before the first anatomy exam at T1 (Mdn (Females) = 8.25 nmol/l; Mdn (Males) = 8.99 nmol/l; U = 735.5, p = 0.1503), there were higher levels in the males at T3, immediately before the second exam (Mdn (Females) = 8.99 nmol/l; Mdn (Males) = 10.90 nmol/l; U = 658.5, p = 0.0389).

### The deep learning approach prevailed

**Fig 2:**
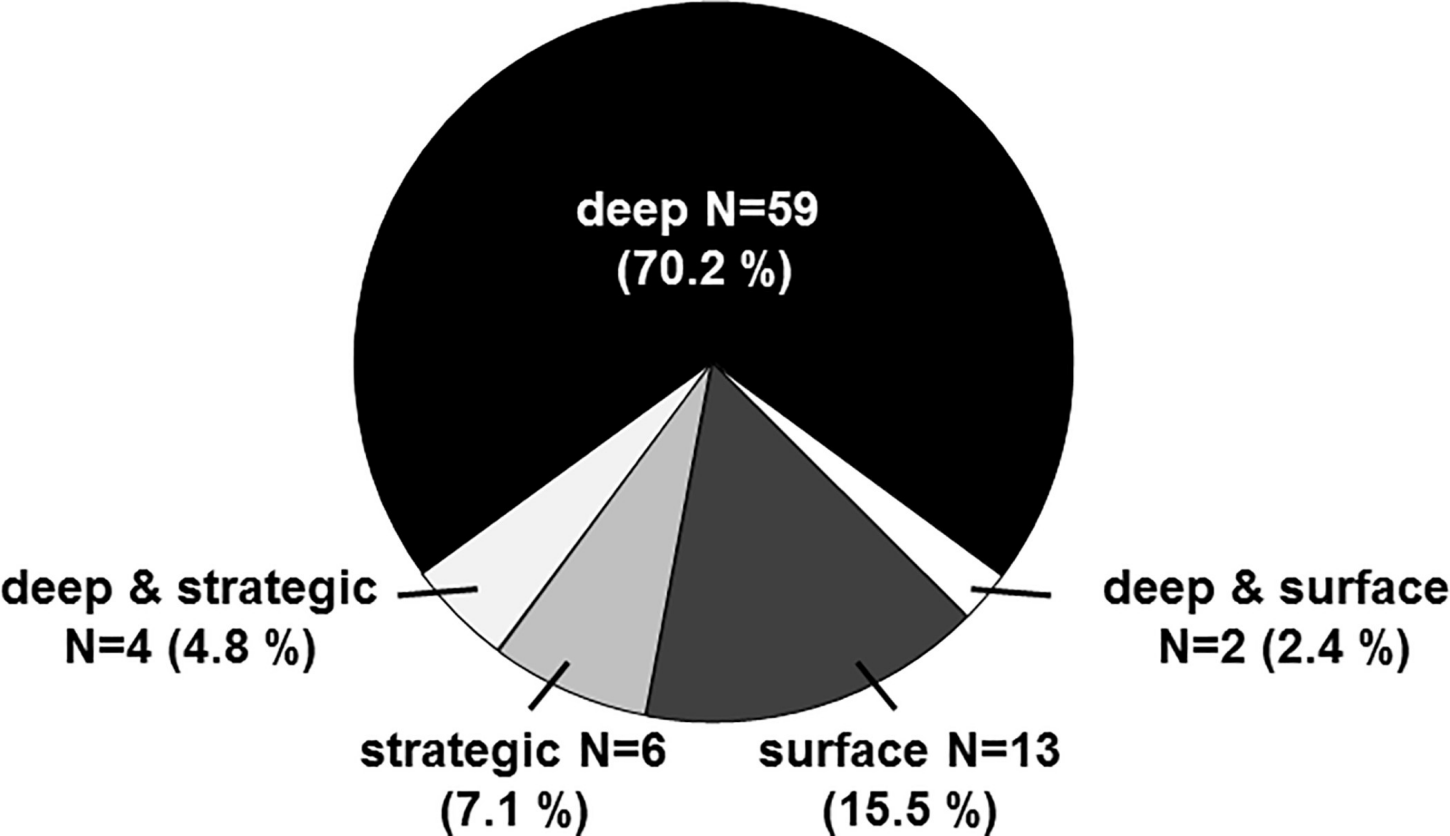
The Deep learning approach prevailed. The pie diagram shows the distribution of learning approaches in our study cohort.

We next assessed the different learning approaches in our study cohort using the ASSIST questionnaire. Of the 84 students who filled out the questionnaire, 59 (70.2%) displayed a predominantly deep learning approach, 6 students (7.1%) displayed a predominantly strategic and 13 (15.5%) a predominantly surface learning approach. Two students combined deep and surface and four combined deep and strategic approaches. This distribution of learning types is illustrated in Fig 2. Types of learning approaches were neither related to gender nor age.

### Surface learning approach correlated with anxiety

**Fig 3:**
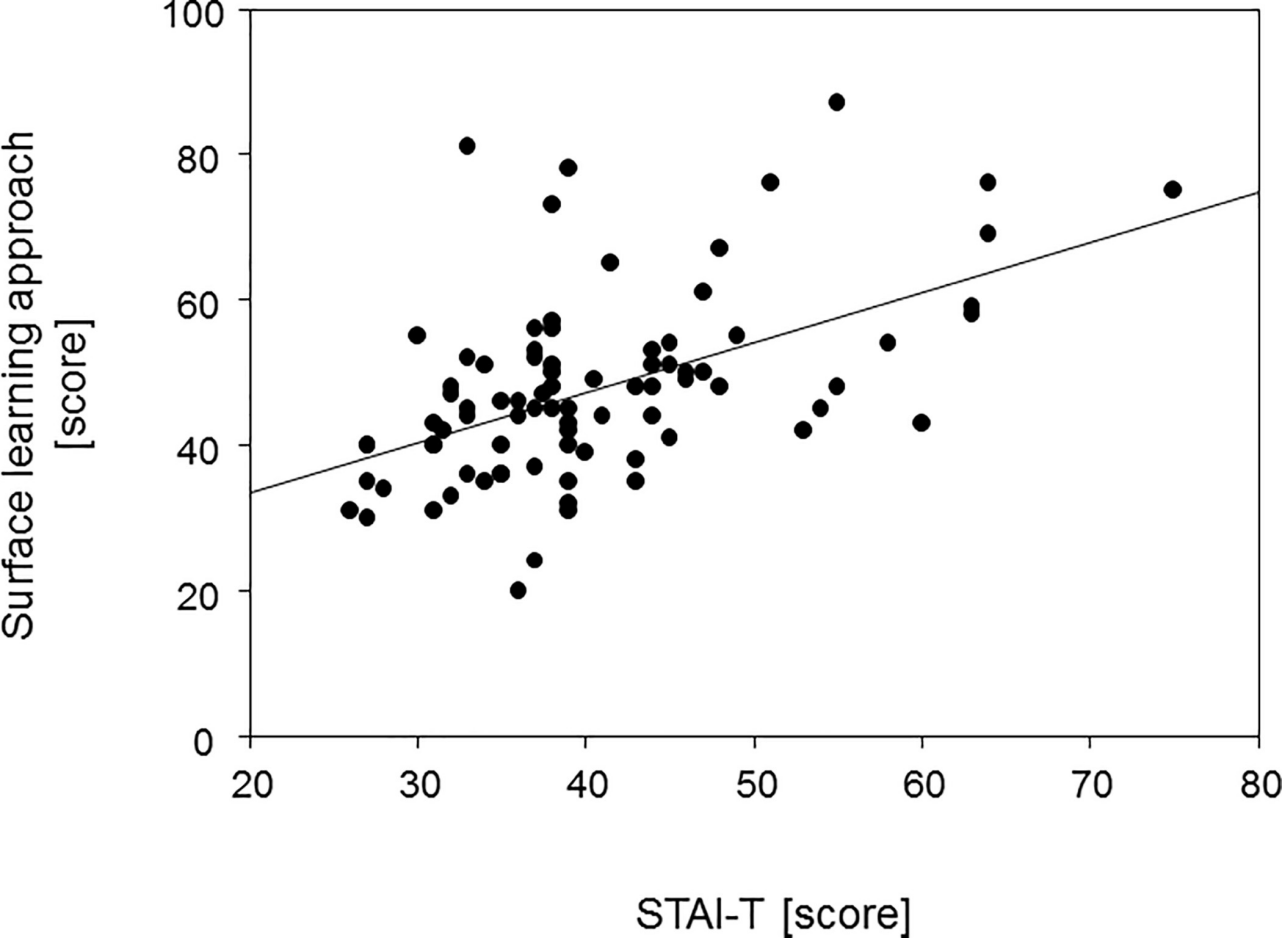
The Surface learning approach correlated significantly with anxiety. The diagram correlates individual scores for the surface learning approach with the corresponding score for trait anxiety. Spearman correlation resulted in a correlation coefficient rs = 0.5026.

We finally set out to investigate whether anxiety was related to the individual learning approach. To that extent we performed spearman rank correlation analyses between the various STAI scores and the individual scores achieved for the deep, strategic and surface learning approaches, respectively. Of note, there was a significant positive correlation between the scores for surface learning and anxiety as a trait (rs(82) = 0.503, p < 0.0001). This correlation is shown in Fig 3. Moreover, there were minor positive correlations between the surface learning approach and exam related anxieties at T1 (rs(82) = 0.212, p = 0.0529) and T3 (rs(82) = 0.2416, p = 0.0268), with significance only being reached at T3. Interestingly, there was also a positive correlation between exam related anxiety at T1 and the corresponding cortisol levels (rs(96) = 0.3387, p = 0.0006) while at T3 significance was not reached (rs(96) = 0.06613, p = 0.5176). Salivary cortisol concentrations did not correlate with a surface learning approach, neither at T1 (rs(82) = -0.03817, p = 0.7303), nor at T3 (rs(82) = -0.03915, p = 0.7236). Likewise, there were no correlations between the deep or strategic learning approaches and either self-reported anxiety (r(82) = -0.07468, p = 0.4996 for deep and r(82) = 0.03297, p = 0.7659 for strategic) or salivary cortisol concentrations (r(82) = 0.02176, p = 0.8442 for deep and r(82) = -0.1137, p = 0.3029 for strategic).

### Students with a strategic approach to learning were the least anxious and academically most successful

**Fig 4:**
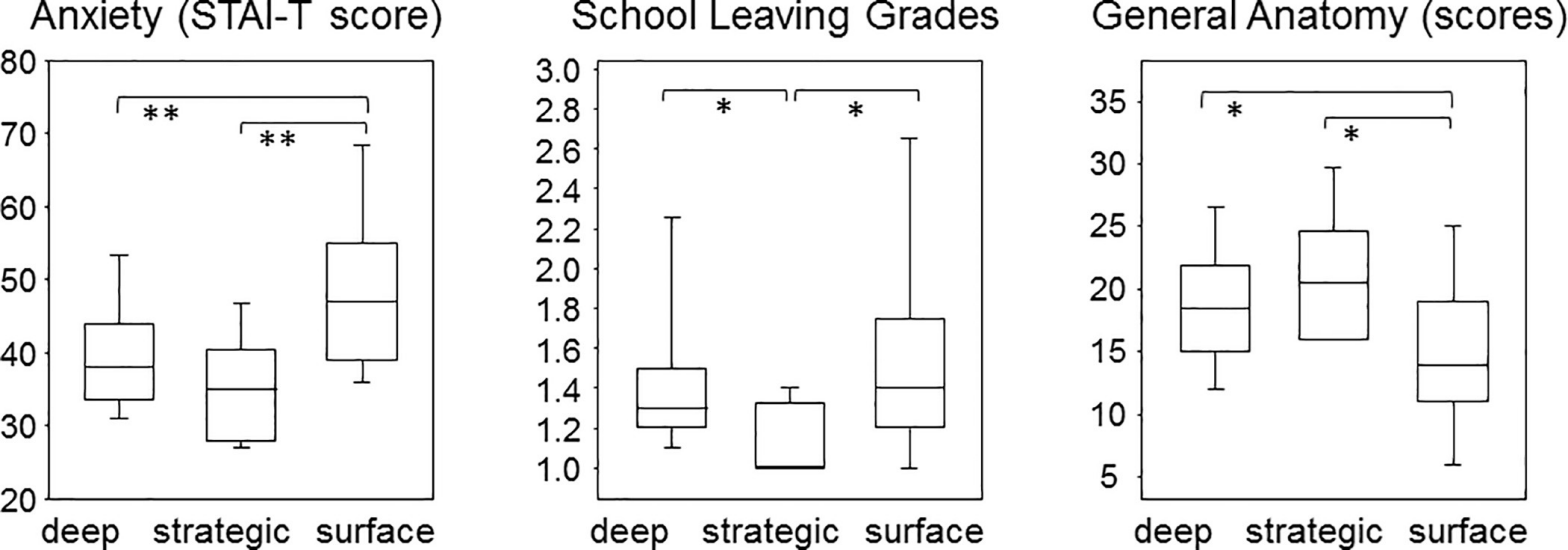
Students with a strategic approach to learning were the least anxious and academically most successful. Box plots compare the scores for trait anxiety, school leaving grades and results from a written general anatomy exam among students with a deep, strategic and surface approach to learning. Statistical significance results from Kruskal-Walis tests (STAI-T and school leaving grades) and ANOVA (general anatomy).

Fig 4 compares the different learning strategies to anxiety-as-a-trait scores and selected academic achievements. There was a statistically significant difference between the trait anxiety scores by different learning strategies (H(2) = 13.3, p = 0.0013) with a mean rank of 42.9 for the deep, 31.1 for the strategic and 66.3 for the surface learners. As the oral anatomy exams used for assessing exam related anxiety were formative and resulted in pass or fail results only, we resorted to both, school leaving grades and test scores achieved during a written general anatomy exam taken during the very first term. The middle and left panels in Fig 4 show that students with a strategic approach to learning yield the best results (note that in Germany the lower school grades are the better) while the poorest results are obtained by students with a surface approach. There were statistically significant differences between the school leaving grades ((H(2) = 8.9, p = 0.0115) with mean ranks of 44.5 for the deep, 19.2 for the strategic and 49.9 for the surface learners) and between the general anatomy scores by learning strategy (F(2, 86) = 4.48, p = 0.014), respectively. Anxiety as a trait neither correlated with school leaving grades (rs(90) = 0.1511, p = 0.1506) nor with the test scores achieved during the written general anatomy exam (rs(94) = -0.11, p = 0.2887).

## Discussion

### Medical students do not seem to give in to constant stress levels

In the present study we did not detect a significant rise in exam related anxiety during the first undergraduate year at medical school. Our data thus contrast previous observations showing that unpleasant exam experiences, high work load and pressure to perform can lead to an increase in exam related anxiety [9]. We here rather assume that the students adapt to the examination process and anxiety levels therefore remain stable. However, exam related anxiety did not decrease either, ruling out the setting in of indifference. Moreover, our results confirm previous work showing that gender had no influence on trait anxiety yet significantly impacted on acute exam related anxiety [19, 8].

### Salivary cortisol confirms self-reported anxiety

We here verified that self-reported anxiety confirmed sympathetic stress parameters. Cortisol measurements were amply shown to be valid and reliable [20] and taking salivary samples is considered even superior to blood samples as it prevents falsification of stress levels due to blood drawing [21]. However, even the salivary sampling is time and resource consuming compared to paper pencil questionnaires. We here showed that salivary cortisol mirrored the self-reported stress as both increased significantly before an exam. Moreover, salivary cortisol and exam related anxieties at T1 significantly correlated with each other and trended towards correlation at T3. We therefore conclude that self-reported exam related anxiety yields comparable results to measuring sympathetic stress parameters and is a reliable means to collect data on acute stress.

### Deep learners predominate among first year medical students

The frequency of learning approaches in our cohort was 70.2% of deep learners, followed by 15.5% of surface and 7.1% of strategic learners. Our results thus confirm previous reports showing that among first year medical students, the deep learning approach predominates [22, 14]. These reports also showed that the frequency of deep learning approaches was higher in medical students compared to other student groups like nurses or dental medicine students and this was attributed to a higher intrinsic motivation among medical students. It was further assumed that the deep learning approach had been adopted already prior to university entry and that selection for highest school grades as admission to medical school favors this particular approach [22]. Our results suggest though, that students with a strategic learning approach achieved even better school grades and were also academically more successful in the first year at medical school, at least in anatomy. The question remains whether students adopt different learning approaches while they progress through medical education or whether a once adopted approach will persist, as several studies already suggested [23, 24]. It will also remain to be investigated which one of the learning approaches will prove most successful during the clinical stages of medical school.

### Anxiety correlates with the surface learning approach

We here showed that anxiety correlates with a surface learning approach. The surface learner is characterized by aiming at minimal requirements, learning implies reproductions and rote learning and as a consequence, difficulties arise in grasping context [12]. Indeed, the surface learning approach could previously be correlated with lowest scores in a high stakes clinical performance examination including patient-physician interaction [25]. Of note, the correlation between anxiety and a surface learning approach does not seem to be restricted to medical students but was also reported for sports students [26].

We consider it likely that exam related anxiety and an anxious personality may reinforce learning difficulties. Students who are influenced by their worries and fear of failure may not be able to structure their learning schedule or focus on the learning material at hand and therefore adopt a surface learning approach [11]. The surface learner in turn is unable to relate bits of information, easily despairs of the limited learning process and therefore loses self-confidence which may reinforce an unfavorable learning intention [12]. However, the chain of events delineated above may also be assembled vice versa with the surface learning approach leading to anxiety. It is reasonable to assume that students who are aware of their adverse learning approaches realize their shortcomings and build up increased anxiety levels. A previous meta-analysis showed that anxiety measured directly after a test performance correlated better with poor performance than anxiety measured before because it is the test outcome that dictates the level of anxiety [27]. While anxiety and surface learning approach may mutually influence each other, our experimental set up does not allow for a clear delineation and calls for further studies in order to elucidate this relationship and possibly identify further factors of influence.

The limitations of our study are the small sample size of our cohort, even smaller subgroups for the various learning styles and implementation at only one medical school. However, our results have broader implications. If indeed a causal relationship between learning approach and anxiety does exist, then fostering a deep learning approach may support the students´ mental health and likewise, conveying strategies to cope with anxiety may increase their academic success.

## Supporting information

S1 Table Text anxiety. (PDF)
