# Supplementary material for: Testing anxiety in undergraduate medical students and its correlation with different learning approaches
Source: PLoS One. 2019 Mar 13;14(3):e0210130. doi: 10.1371/journal.pone.0210130 (PMC6415780; doi:10.1371/journal.pone.0210130)
Supplement: S1 Table — (PDF) [file pone.0210130.s001.pdf]

student	STAI-T (T0)	STAI-S (T1)	STAI-S (T3)	Cortisol (T1) nmol/l	Cortisol (T2) nmol/l	Cortisol (T3) nmol/l	Cortisol (T4) nmol/l	school leaving grade	SPF (score)
1	41	55	54	15.90	6.93	11.70	10.00	2.7	41
2	63	51	43	6.01	5.56	4.34	1.50	1.0	49
3	32	28	26	7.02	8.62	8.85	10.30	2.5	
4	43	52	39	4.69	3.13	7.75	3.97	1.4	41
5	37	54	55	6.70	4.56	6.26	5.56	1.7	49
6	31	65	66	10.50	4.96	14.70	3.07	2.5	34
7	39	57	42	7.86	7.28	19.40	9.52	1.0	45
8	42	58	60	7.16	5.03	9.76	7.30	1.5	41
9	60	58	60	11.20	5.22	8.44	2.40	1.6	
10	43	70	67	14.40	3.54	15.50	6.84	2.7	45
11	27	45	33	6.68	4.86	7.27	4.38	1.0	42
12	48	54	59	15.60	8.28	19.60	14.70	2.5	40
13	33	40	57	6.74	1.96	10.60	5.84	1.2	48
14	58	49	48	5.08	9.04	11.70	5.25	2.7	
15	36	37	38	6.91	21.50	16.70	15.40	1.0	42
16	75	67	69	12.30	12.30	12.90	10.00	2.5	55
17	31	52	53	9.60	7.48	4.81	7.57	1.0	52
18	30	74	71	13.80	8.92	15.70	6.06	2.8	42
19	35	43	48	7.30	9.39	6.31	5.82	1.8	34
20	44	46	52	5.71	13.50	8.20	5.65	1.0	47
21	33	53	48	8.45	4.10	7.27	20.10	1.0	39
22	34	48	45	5.71	5.00	9.66	10.40	1.0	
23	47	51	63	5.51	5.61	6.93	6.63	2.8	44
24	26	36	37	4.50	5.32	4.72	4.17	1.4	40
25	38	55	53	8.13	6.95	11.60	6.76	1.3	39
26	41	63	57	8.17	5.18	10.40	6.74	1.1	44
27	34	47	53	8.57	6.88	11.40	10.20	1.0	52
28	39	36	47	13.80	11.10	8.21	6.42	1.9	
29	33	53	51	9.13	10.00	11.90	8.39	1.0	40
30	37	55	46	13.20	11.60	5.13	7.01	1.0	45
31	45	70	59	19.10	4.70	8.51	5.63	1.3	44
32	45	49	56	8.99	4.32	5.77	4.76	1.1	36
33	34	43	50	9.27	3.26	10.20	3.69	1.3	41
34	53	63	44	9.59	3.02	4.72	14.40	1.1	39
35	38	67	67	6.89	21.30	12.70	4.47	1.5	33
36	39	48	49	4.92	7.68	13.00	8.50	1.1	57

37	44	56	65	4.87	4.41	8.58	3.34	1.3	44
38	38	58	64	11.20	5.60	8.50	12.50	1.6	51
39	33	39	50	11.10	6.39	6.23	15.60	1.4	44
40	39	58	55	10.30	4.70	22.50	28.30	1.2	44
41	31	59	48	14.70	17.70	9.40	4.08	1.5	50
42	55	46	61	6.64	7.99	4.34	3.98	1.4	35
43	32	59	40	12.60	9.51	12.70	13.50	1.2	40
44	31	46	51	5.84	4.74	4.73	4.98	1.1	40
45	40	56	59	9.17	12.60	9.94	7.12	1.1	
46	61	69	76	3.68	4.51	5.01	5.30	1.5	
47	46	59	65	7.06	3.13	4.63	2.70	1.5	41
48	45	49	73	5.94	9.49	13.10	9.04	1.4	51
49	49	55	56	9.27	3.74	3.88	7.36	1.4	49
50	27	33	39	4.92	4.39	8.95	4.80	1.5	50
51	33	55	60	8.28	9.76	8.99	9.86	1.4	42
52	36	63	72	10.90	13.70	14.00	2.76		
53	51	64	70	10.90	9.11	10.40	11.60	1.4	46
54	44	55	54	24.60	10.70	8.88	4.65	1.4	48
55	48	53	65	6.36	9.36	5.96	4.54	1.4	46
56	32	65	65	23.80	7.71	15.50	2.05	1.4	38
57	39	44	39	7.31	8.43	8.49	11.80	1.1	42
58	35	44	38	14.40	13.20	9.04	11.50	1.2	37
59	64	62	71	6.66	7.54	10.70	4.02	1.2	44
60	55	44	60	7.44	2.93	25.10	9.52	1.5	42
61	44	46	67	6.08	6.07	5.56	2.44	1.3	42
62	67	49	51	4.87	16.20	18.60	12.90	1.3	47
63	33	48	55	7.34	9.67	21.20	11.30	1.4	40
64	28	55	51	20.70	2.30	9.18	7.86	1.0	37
65	54	68	54	9.00	6.42	11.60	7.70	1.2	54
66	32	74	68	9.94	7.23	9.33	4.80	1.2	44
67	64	43	42	7.36	8.96	11.30	8.59	1.2	47
68	37	57	60	5.22	4.65	6.31	6.46	1.4	
69	38	65	44	25.40	12.80	21.40	2.75		
70	38	42	45	11.20	5.63	10.90	8.07	1.2	48
71	39	54	64	7.00	4.17	6.99	3.04	1.3	44
72	38	44	51	8.47	8.61	27.60	15.30	1.4	42
73	36	44	52	16.20	15.40	6.77	7.92	1.1	45
74	35	48	50	20.10	4.40	14.10	6.76	1.3	
75	27	39	39	4.77	5.05	6.43	4.88		35

76	27	57	63	6.40	5.73	7.39	6.09	1.5	48
77	39	68	36	7.43	5.86	6.86	7.59		38
78	45	47	51	6.41	4.61	20.70	18.90	1.0	49
79	39	50	55	7.81	4.72	12.20	8.86	1.4	42
80	36	64	66	10.50	5.12	8.63	9.45	1.2	44
81	35	38	48	13.10	10.80	16.60	9.08	1.2	46
82	38	54	56	15.40	4.61	9.91	11.90	1.2	41
83	43	45	50	7.69	4.04	17.80	4.04	1.3	46
84	36	42	40	6.68	9.88	15.00	5.37	1.2	51
85	63	71	53	7.40	8.87	5.02	3.66	1.0	45
86	40	51	52	11.20	9.15	13.30	7.32	1.3	49
87	37	75	77	15.30	4.69	28.70	4.17	1.1	
88	46	45	45	5.18	6.49	5.55	12.20	1.6	50
89	47	61	61	8.23	4.97	10.10	5.75	1.4	39
90	50	56	61	11.50	14.90	13.30	12.00	1.4	43
91	37	29	51	8.25	10.40	5.71	5.49	1.1	34
92	38	63	61	16.30	6.13	6.75	4.99	1.5	45
93	45	61	61	14.50	20.80	8.80	7.57	1.6	44
94	41	51	45	15.00	2.28	32.30	3.70	1.6	42
95	37	53	54	20.10	5.14	28.70	5.14		
96	37	55	55	12.00	5.79	10.00	6.62	1.6	43
97	41	63	55	8.19	7.85	6.15	6.38	1.8	47
98	38	58	49	10.30	7.23	8.31	6.42		37

sex	predominant learning approach	deep (score)	strategic (score)	surface (score)	age (years)	written anatomy exam (scores)
male	deep	73	63	49	30	16
female	deep	63	56	59	21	26
male	deep	62	65	42	29	16
female	surface	45	35	48	22	19
female	deep	66	66	56	24	
female	deep	58	54	40	28	11
female	surface	58	78	78	21	20
female	surface	44	41	65	21	13
female	deep	61	50	43	27	17
female	deep	53	47	38	28	12
female	strategic	65	93	40	22	30
male	surface	63	61	67	28	14
female	deep	57	47	52	23	22
female	deep	63	67	54	29	17
female					21	29
female	surface	63	71	75	21	6
female	deep	69	49	43	21	19
female	deep	64	60	55	20	13
male	deep	53	53	40	21	18
female	deep	68	60	51	20	20
female	surface	62	81	81	21	6
female	deep /	51	63	35	22	27
female	surface	59	61	61	29	11
female	deep	64	57	31	20	17
female	deep	60	67	51	24	22
female	deep	52	53	44	21	24
female					22	19
male	deep	62	54	45	24	21
female					21	22
female	strategic	56	74	53	22	21
female	deep	65	69	51	20	23
male					22	15
female	deep	61	69	51	24	29
female	deep	64	60	42	22	14
male	surface	54	73	73	22	23
female	deep	75	61	31	20	14

female	deep	58	66	44	20	24
female	deep	62	55	45	22	20
female	deep	58	59	45	22	22
male	deep	72	50	32	21	24
male	deep	58	53	31	22	15
male	dee / surface	48	48	48	22	19
female	deep	51	61	48	21	15
female	deep	65	65	40	22	23
female	deep	59	60	39	20	18
female					23	22
female	deep	71	62	50	21	15
female					22	
female	deep	59	45	55	22	20
female	deep	69	64	35	21	17
female	deep	57	50	44	20	20
female					21	
female	surface	63	76	76	20	14
female	deep	63	65	48	23	30
female	deep	67	62	48	22	17
female	strategic	50	78	33	21	16
male	deep	62	58	43	21	22
female	deep	58	58	46	22	20
female	surface	58	50	76	21	13
male	surface	59	87	87	22	28
female	deep / surface	53	44	53	21	9
female					21	28
female	deep	70	59	36	23	31
male	strategic	66	86	34	21	16
female	deep	68	78	45	20	14
female	deep	51	60	47	20	14
female	surface	52	69	69	20	16
female	deep	61	50	52	22	22
female					21	27
male	deep	61	55	48	22	16
female	deep	65	66	42	20	17
male	deep	64	46	50	20	14
female	deep /strategic	48	60	20	21	22
female	deep	59	71	36	21	25
female	deep /	52	65	40	21	19

female	deep	60	71	30	20	11
male	strategic	48	75	40	21	20
male	strategic	66	87	41	21	24
female	deep	62	58	35	21	11
female	deep	67	55	44	21	27
male	deep	76	66	36	20	27
male	deep	67	69	47	21	19
male	deep	74	68	35	21	11
male	deep	56	52	46	20	21
female	deep	73	50	58	21	15
female					21	25
female					20	10
male	deep	64	61	49	22	16
female	deep /	54	67	50	20	16
female					21	20
female	deep	61	55	37	22	23
female	deep	71	51	56	20	12
female	deep	64	72	54	20	16
male					23	13
male	deep	67	74	45	20	15
male	deep	57	57	24	23	23
female					21	22
female	surface	51	46	57	21	16
